# Bis[(1,1′-biphenyl-2,2′-di­yl)di-*tert*-butyl­phospho­nium] di-μ-chlorido-bis­[dichlorido­palladate(II)]

**DOI:** 10.1107/S1600536812037786

**Published:** 2012-09-08

**Authors:** Charmaine Arderne, Cedric W. Holzapfel

**Affiliations:** a Department of Chemistry, University of Johannesburg, P O Box 524, Auckland Park, Johannesburg, 2006, South Africa

## Abstract

In the title compound, (C_20_H_26_P)_2_[Pd_2_Cl_6_], the Pd^II^ atom within the hexachloridodipalladate(II) dianion has a square-planar geometry. It resides on a centre of inversion with the asymmetric unit containing half of the dianion and one phospho­nium cation. Only weak C—H⋯π inter­actions are present in the crystal structure.

## Related literature

For the structures of related Pd_II_ complexes and background to organopalladium-catalysed reactions, see: Ormondi *et al.* (2011 ▶); Williams *et al.* (2008 ▶); Migowski & DuPont (2007 ▶); d′OrLyé & Jutland (2005 ▶); Beletskaya & Cheprakov (2004 ▶). For a description of the Cambridge Structural Database, see: Allen (2002 ▶).
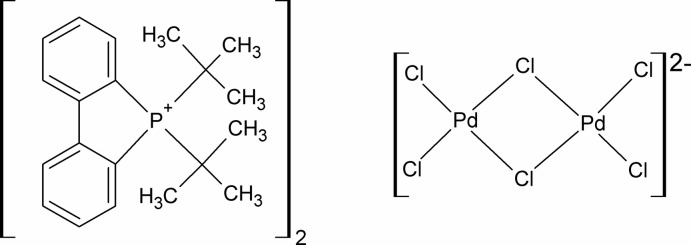


## Experimental

### 

#### Crystal data

(C_20_H_26_P)_2_[Pd_2_Cl_6_]
*M* *_r_* = 1020.26
Triclinic, 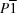

*a* = 8.3247 (2) Å
*b* = 11.2697 (2) Å
*c* = 11.7004 (3) Å
α = 73.0982 (6)°
β = 85.0900 (6)°
γ = 82.2708 (5)°
*V* = 1039.49 (4) Å^3^
*Z* = 1
Mo *K*α radiation
μ = 1.36 mm^−1^
*T* = 100 K
0.29 × 0.22 × 0.20 mm

#### Data collection

Bruker APEXII CCD diffractometer
Absorption correction: multi-scan (*AXScale*; Bruker, 2010 ▶) *T*_min_ = 0.695, *T*_max_ = 0.774
39747 measured reflections
5184 independent reflections
5088 reflections with *I* > 2σ(*I*)
*R*_int_ = 0.017

#### Refinement

*R*[*F* ^2^ > 2σ(*F* ^2^)] = 0.015
*wR*(*F* ^2^) = 0.037
*S* = 1.05
5184 reflections
232 parameters
H-atom parameters constrained
Δρ_max_ = 0.41 e Å^−3^
Δρ_min_ = −0.48 e Å^−3^

### 

Data collection: *APEX2* (Bruker, 2010 ▶); cell refinement: *SAINT* (Bruker, 2010 ▶); data reduction: *SAINT*; program(s) used to solve structure: *SHELXS97* (Sheldrick, 2008 ▶); program(s) used to refine structure: *SHELXL97* (Sheldrick, 2008 ▶); molecular graphics: *OLEX2* (Dolomanov *et al.*, 2009 ▶); software used to prepare material for publication: *publCIF* (Westrip, 2010 ▶) and *PLATON* (Spek, 2009 ▶).

## Supplementary Material

Crystal structure: contains datablock(s) I, global. DOI: 10.1107/S1600536812037786/gg2094sup1.cif

Structure factors: contains datablock(s) I. DOI: 10.1107/S1600536812037786/gg2094Isup2.hkl

Supplementary material file. DOI: 10.1107/S1600536812037786/gg2094Isup3.mol

Additional supplementary materials:  crystallographic information; 3D view; checkCIF report

## Figures and Tables

**Table 1:** Hydrogen-bond geometry (Å, °) *Cg*7 is the centroid of the Pd1,Cl3,Pd1′,Cl3′ ring.

*D*—H⋯*A*	*D*—H	H⋯*A*	*D*⋯*A*	*D*—H⋯*A*
C11—H11⋯*Cg*7^i^	0.95	2.68	3.5952 (13)	138

Symmetry code: (i) 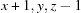.

**Fig. 1.**
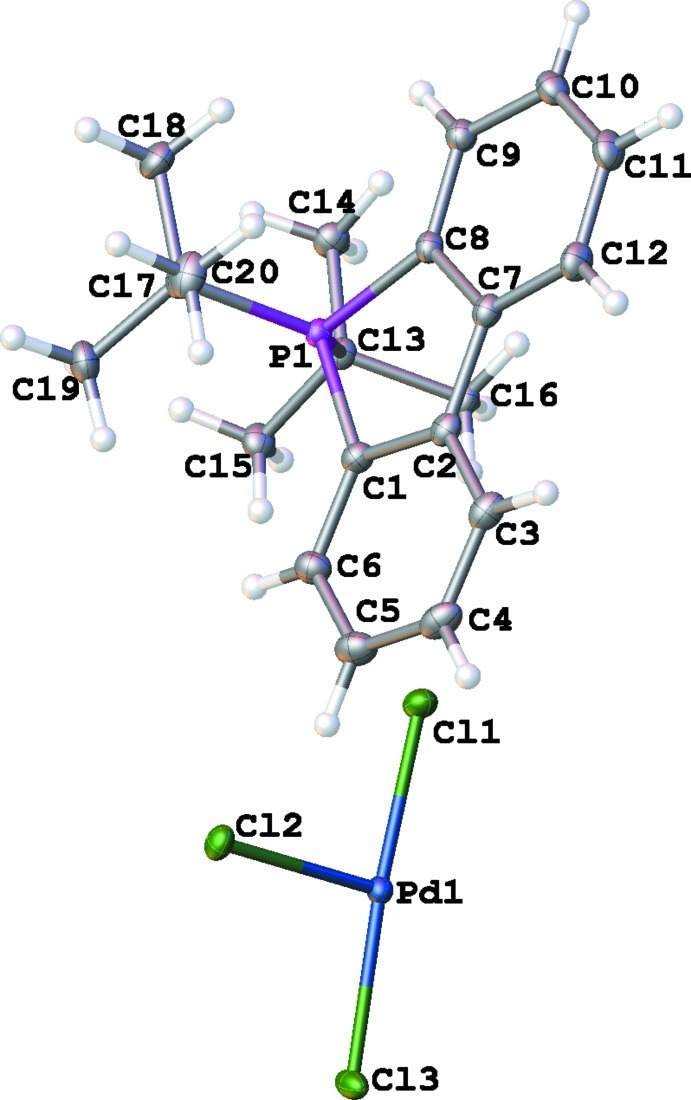
: Molecular structure of the asymmetric unit of the title compound (I) with thermal displacement ellipsoids drawn at the 50% probability level.

## supplementary crystallographic information

### Comment

As part of our continued studies (Williams *et al.*, 2008 and Ormondi *et al.*, 2011) of organopalladium catalysed reactions, we have found that certain palladocycles (Beletskaya & Cheprakov, 2004 and d'OrLyé & Jutland, 2005) are readily converted into highly catalytically active low-ligated Pd^0^ complexes. We now report that treatment of one such palladocycle, namely acetato-(2'-di-*t*-butylphosphino-1,1'-diphenyl-2yl) palladium(II), with HCl at room temperature results in the formation of the title compound (I) in good yield. Formation of the complex appears to result from the acid-induced reductive elimination of Pd^0^ from the palladocycle followed by oxidation of the palladium in the presence of air and chloride ions (Migowski & DuPont, 2007).

The structure of the title compound (I), [C_20_H_26_P. 0.5(Cl_6_Pd_2_)]_2_ shows a square planar geometry for the Pd^II^ atom within the hexachlorodipalladium(II) anion. The palladium atom sits on a centre of inversion and therefore the asymmetric unit contains half of the trichloropalladium(II) anion and one phosphonium cation. Figure 1 shows a diagram of the molecular structure of the asymmetric unit of (I). Weak interactions were observed in this structure where C—H···Cl and C—H···π are evident only.

### Experimental

A solution of hydrogen chloride (142 mg; 4 mmol) in 18 ml of methanol was slowly added to a stirred solution of the palladocycle precursor, namely acetato-(2'-di-*t*-butylphosphino-1,1'-diphenyl-2yl) palladium(II) (493 mg; 1 mmol) in 17 ml of dichloromethane over a period of 10 minutes. The reaction mixture changed from colourless to dark purple and then to dark brown. After completion of the addition, stirring was discontinued and the reaction mixture left exposed to the air at room temperature. A red crystalline precipitate started to form after 45 minutes. After 24 h, the supernatant solution was removed, the solid material was with ether and dried *in vacuo*. The solid material (376 mg; 74%) was taken up in 15 ml of 2:1 dichloromethane:methanol and the resulting solution was exposed to the vapours of diethyl ether in a closed system for 24 h. Well formed, dark red prisms of the title compound (I) crystallized from the solution and a suitable single-crystal was selected for the single-crystal X-ray diffraction analysis.

### Refinement

The H-atoms were geometrically positioned and refined in the riding-model approximation, with C—H = 0.97 Å, N—H = 0.89 Å, and *U*_iso_(H) = 1.2Ueq(C) or 1.5Ueq(N). For (I), the highest peak in the final difference map is 0.60 Å from Cl4B and the deepest hole is 0.27 Å from Cl4B.

### Crystal data

**Table:**

(C_20_H_26_P)_2_[Pd_2_Cl_6_]	*Z* = 1
*M**_r_* = 1020.26	*F*(000) = 516
Triclinic, *P*1	*D*_x_ = 1.630 Mg m^−^^3^
Hall symbol: -P 1	Mo *K*α radiation, λ = 0.71073 Å
*a* = 8.3247 (2) Å	Cell parameters from 9253 reflections
*b* = 11.2697 (2) Å	θ = 3.6–28.4°
*c* = 11.7004 (3) Å	µ = 1.36 mm^−^^1^
α = 73.0982 (6)°	*T* = 100 K
β = 85.0900 (6)°	Prism, dark orange
γ = 82.2708 (5)°	0.29 × 0.22 × 0.20 mm
*V* = 1039.49 (4) Å^3^	

### Data collection

**Table:**

Bruker APEXII CCD diffractometer	5184 independent reflections
Radiation source: fine-focus sealed tube	5088 reflections with *I* > 2σ(*I*)
Graphite monochromator	*R*_int_ = 0.017
φ and ω scans	θ_max_ = 28.5°, θ_min_ = 2.9°
Absorption correction: multi-scan (AXScale; Bruker, 2010)	*h* = −11→11
*T*_min_ = 0.695, *T*_max_ = 0.774	*k* = −15→15
39747 measured reflections	*l* = −15→15

### Refinement

**Table:**

Refinement on *F*^2^	Primary atom site location: structure-invariant direct methods
Least-squares matrix: full	Secondary atom site location: difference Fourier map
*R*[*F*^2^ > 2σ(*F*^2^)] = 0.015	Hydrogen site location: inferred from neighbouring sites
*wR*(*F*^2^) = 0.037	H-atom parameters constrained
*S* = 1.05	*w* = 1/[σ^2^(*F*_o_^2^) + (0.0139*P*)^2^ + 0.6282*P*] where *P* = (*F*_o_^2^ + 2*F*_c_^2^)/3
5184 reflections	(Δ/σ)_max_ = 0.002
232 parameters	Δρ_max_ = 0.41 e Å^−^^3^
0 restraints	Δρ_min_ = −0.48 e Å^−^^3^

### Special details

**Table:**

Geometry. All e.s.d.'s (except the e.s.d. in the dihedral angle between two l.s. planes) are estimated using the full covariance matrix. The cell e.s.d.'s are taken into account individually in the estimation of e.s.d.'s in distances, angles and torsion angles; correlations between e.s.d.'s in cell parameters are only used when they are defined by crystal symmetry. An approximate (isotropic) treatment of cell e.s.d.'s is used for estimating e.s.d.'s involving l.s. planes.
Refinement. Refinement of *F*^2^ against ALL reflections. The weighted *R*-factor *wR* and goodness of fit *S* are based on *F*^2^, conventional *R*-factors *R* are based on *F*, with *F* set to zero for negative *F*^2^. The threshold expression of *F*^2^ > σ(*F*^2^) is used only for calculating *R*-factors(gt) *etc*. and is not relevant to the choice of reflections for refinement. *R*-factors based on *F*^2^ are statistically about twice as large as those based on *F*, and *R*- factors based on ALL data will be even larger.

### Fractional atomic coordinates and isotropic or equivalent isotropic displacement parameters (Å^2^)

**Table:**

	*x*	*y*	*z*	*U*_iso_*/*U*_eq_	
C16	0.06541 (14)	0.17413 (10)	0.35252 (11)	0.0171 (2)	
H16A	0.1167	0.1453	0.4297	0.026*	
H16B	0.1288	0.1356	0.2953	0.026*	
H16C	−0.0451	0.1504	0.3628	0.026*	
C2	0.51131 (13)	0.18374 (10)	0.38452 (10)	0.0150 (2)	
C6	0.36510 (14)	0.28059 (11)	0.52998 (11)	0.0182 (2)	
H6	0.2787	0.3363	0.5501	0.022*	
C1	0.38564 (13)	0.26782 (10)	0.41465 (10)	0.0147 (2)	
C3	0.61953 (14)	0.11512 (11)	0.47087 (11)	0.0186 (2)	
H3	0.7057	0.0588	0.4515	0.022*	
C5	0.47340 (15)	0.21017 (12)	0.61544 (11)	0.0213 (2)	
H5	0.4601	0.2173	0.6947	0.026*	
C4	0.60052 (15)	0.12966 (12)	0.58561 (11)	0.0215 (2)	
H4	0.6753	0.0840	0.6441	0.026*	
C17	0.32342 (14)	0.50983 (10)	0.22795 (11)	0.0161 (2)	
C13	0.05899 (13)	0.31677 (10)	0.30507 (10)	0.0137 (2)	
C7	0.51087 (13)	0.17674 (10)	0.26061 (10)	0.0149 (2)	
C19	0.25132 (15)	0.58473 (11)	0.31437 (12)	0.0215 (2)	
H19A	0.2870	0.5412	0.3951	0.032*	
H19B	0.1326	0.5930	0.3149	0.032*	
H19C	0.2883	0.6678	0.2884	0.032*	
C18	0.26103 (15)	0.57096 (11)	0.10206 (11)	0.0211 (2)	
H18A	0.1421	0.5790	0.1061	0.032*	
H18B	0.3034	0.5190	0.0496	0.032*	
H18C	0.2977	0.6539	0.0701	0.032*	
C15	−0.03314 (14)	0.37709 (11)	0.39742 (11)	0.0181 (2)	
H15A	−0.0455	0.4681	0.3642	0.027*	
H15B	0.0279	0.3537	0.4702	0.027*	
H15C	−0.1406	0.3479	0.4167	0.027*	
C12	0.61554 (14)	0.09816 (11)	0.20828 (11)	0.0190 (2)	
H12	0.7014	0.0445	0.2517	0.023*	
C14	−0.02701 (14)	0.36283 (11)	0.18602 (11)	0.0180 (2)	
H14A	0.0365	0.3286	0.1260	0.027*	
H14B	−0.0369	0.4542	0.1584	0.027*	
H14C	−0.1353	0.3350	0.1979	0.027*	
C20	0.50976 (14)	0.50237 (12)	0.22269 (13)	0.0228 (2)	
H20A	0.5562	0.4503	0.1713	0.034*	
H20B	0.5491	0.4656	0.3034	0.034*	
H20C	0.5426	0.5865	0.1900	0.034*	
C9	0.36534 (14)	0.25847 (10)	0.07624 (10)	0.0157 (2)	
H9	0.2817	0.3136	0.0314	0.019*	
C8	0.38668 (13)	0.25742 (10)	0.19316 (10)	0.0137 (2)	
C10	0.46880 (14)	0.17729 (11)	0.02614 (11)	0.0183 (2)	
H10	0.4545	0.1754	−0.0530	0.022*	
C11	0.59299 (15)	0.09897 (11)	0.09144 (12)	0.0205 (2)	
H11	0.6639	0.0449	0.0557	0.025*	
Cl3	0.06462 (4)	0.07944 (3)	1.07304 (2)	0.01962 (6)	
Cl2	0.15259 (3)	0.29365 (2)	0.81900 (2)	0.01663 (5)	
Cl1	0.01589 (3)	0.12552 (3)	0.67310 (2)	0.01763 (6)	
P1	0.27397 (3)	0.34725 (3)	0.28353 (2)	0.01179 (5)	
Pd1	0.041384 (9)	0.110164 (7)	0.868729 (7)	0.01193 (3)	

### Atomic displacement parameters (Å^2^)

**Table:**

	*U*^11^	*U*^22^	*U*^33^	*U*^12^	*U*^13^	*U*^23^
C16	0.0169 (5)	0.0132 (5)	0.0208 (5)	−0.0040 (4)	0.0012 (4)	−0.0037 (4)
C2	0.0129 (5)	0.0132 (5)	0.0177 (5)	−0.0035 (4)	−0.0001 (4)	−0.0018 (4)
C6	0.0177 (5)	0.0209 (6)	0.0170 (5)	−0.0038 (4)	−0.0011 (4)	−0.0060 (4)
C1	0.0135 (5)	0.0149 (5)	0.0151 (5)	−0.0025 (4)	−0.0017 (4)	−0.0025 (4)
C3	0.0134 (5)	0.0170 (5)	0.0230 (6)	−0.0027 (4)	−0.0025 (4)	−0.0010 (4)
C5	0.0214 (6)	0.0260 (6)	0.0173 (5)	−0.0072 (5)	−0.0036 (4)	−0.0046 (5)
C4	0.0180 (5)	0.0228 (6)	0.0213 (6)	−0.0059 (4)	−0.0071 (4)	0.0009 (5)
C17	0.0149 (5)	0.0123 (5)	0.0208 (5)	−0.0032 (4)	0.0008 (4)	−0.0042 (4)
C13	0.0118 (5)	0.0128 (5)	0.0162 (5)	−0.0021 (4)	0.0006 (4)	−0.0037 (4)
C7	0.0127 (5)	0.0126 (5)	0.0181 (5)	−0.0020 (4)	0.0003 (4)	−0.0026 (4)
C19	0.0229 (6)	0.0161 (5)	0.0280 (6)	−0.0024 (4)	−0.0005 (5)	−0.0103 (5)
C18	0.0236 (6)	0.0157 (5)	0.0210 (6)	−0.0038 (4)	0.0000 (5)	−0.0001 (4)
C15	0.0161 (5)	0.0174 (5)	0.0204 (6)	−0.0012 (4)	0.0035 (4)	−0.0064 (4)
C12	0.0152 (5)	0.0159 (5)	0.0242 (6)	0.0015 (4)	0.0009 (4)	−0.0049 (5)
C14	0.0151 (5)	0.0192 (5)	0.0193 (5)	−0.0013 (4)	−0.0033 (4)	−0.0043 (4)
C20	0.0151 (5)	0.0189 (6)	0.0337 (7)	−0.0055 (4)	0.0013 (5)	−0.0052 (5)
C9	0.0152 (5)	0.0149 (5)	0.0169 (5)	−0.0023 (4)	0.0007 (4)	−0.0043 (4)
C8	0.0127 (5)	0.0117 (5)	0.0163 (5)	−0.0015 (4)	0.0014 (4)	−0.0039 (4)
C10	0.0193 (5)	0.0188 (5)	0.0184 (5)	−0.0039 (4)	0.0034 (4)	−0.0082 (4)
C11	0.0187 (5)	0.0173 (5)	0.0255 (6)	−0.0001 (4)	0.0045 (4)	−0.0088 (5)
Cl3	0.03037 (15)	0.01788 (13)	0.01368 (12)	−0.01237 (11)	0.00371 (10)	−0.00635 (10)
Cl2	0.01830 (12)	0.01205 (11)	0.01884 (13)	−0.00401 (9)	−0.00206 (10)	−0.00193 (10)
Cl1	0.02204 (13)	0.01686 (12)	0.01351 (12)	−0.00352 (10)	−0.00294 (9)	−0.00237 (10)
P1	0.01112 (12)	0.01095 (12)	0.01294 (12)	−0.00073 (9)	−0.00015 (9)	−0.00320 (10)
Pd1	0.01306 (4)	0.01056 (4)	0.01228 (4)	−0.00235 (3)	0.00107 (3)	−0.00344 (3)

### Geometric parameters (Å, º)

**Table:**

C16—C13	1.5356 (15)	C19—H19C	0.9800
C16—H16A	0.9800	C18—H18A	0.9800
C16—H16B	0.9800	C18—H18B	0.9800
C16—H16C	0.9800	C18—H18C	0.9800
C2—C3	1.3945 (15)	C15—H15A	0.9800
C2—C1	1.4075 (15)	C15—H15B	0.9800
C2—C7	1.4751 (16)	C15—H15C	0.9800
C6—C1	1.3920 (16)	C12—C11	1.3930 (18)
C6—C5	1.3939 (16)	C12—H12	0.9500
C6—H6	0.9500	C14—H14A	0.9800
C1—P1	1.8003 (11)	C14—H14B	0.9800
C3—C4	1.3924 (18)	C14—H14C	0.9800
C3—H3	0.9500	C20—H20A	0.9800
C5—C4	1.3884 (18)	C20—H20B	0.9800
C5—H5	0.9500	C20—H20C	0.9800
C4—H4	0.9500	C9—C8	1.3911 (16)
C17—C19	1.5335 (16)	C9—C10	1.3913 (16)
C17—C18	1.5357 (17)	C9—H9	0.9500
C17—C20	1.5391 (16)	C8—P1	1.7945 (11)
C17—P1	1.8476 (11)	C10—C11	1.3890 (17)
C13—C15	1.5371 (15)	C10—H10	0.9500
C13—C14	1.5404 (15)	C11—H11	0.9500
C13—P1	1.8500 (11)	Cl3—Pd1^i^	2.3166 (3)
C7—C12	1.3908 (16)	Cl3—Pd1	2.3349 (3)
C7—C8	1.4100 (15)	Cl2—Pd1	2.2791 (3)
C19—H19A	0.9800	Cl1—Pd1	2.2709 (3)
C19—H19B	0.9800	Pd1—Cl3^i^	2.3166 (3)
			
C13—C16—H16A	109.5	H18A—C18—H18C	109.5
C13—C16—H16B	109.5	H18B—C18—H18C	109.5
H16A—C16—H16B	109.5	C13—C15—H15A	109.5
C13—C16—H16C	109.5	C13—C15—H15B	109.5
H16A—C16—H16C	109.5	H15A—C15—H15B	109.5
H16B—C16—H16C	109.5	C13—C15—H15C	109.5
C3—C2—C1	119.27 (11)	H15A—C15—H15C	109.5
C3—C2—C7	126.57 (11)	H15B—C15—H15C	109.5
C1—C2—C7	114.15 (10)	C7—C12—C11	119.26 (11)
C1—C6—C5	118.86 (11)	C7—C12—H12	120.4
C1—C6—H6	120.6	C11—C12—H12	120.4
C5—C6—H6	120.6	C13—C14—H14A	109.5
C6—C1—C2	121.02 (10)	C13—C14—H14B	109.5
C6—C1—P1	130.24 (9)	H14A—C14—H14B	109.5
C2—C1—P1	108.73 (8)	C13—C14—H14C	109.5
C4—C3—C2	119.62 (11)	H14A—C14—H14C	109.5
C4—C3—H3	120.2	H14B—C14—H14C	109.5
C2—C3—H3	120.2	C17—C20—H20A	109.5
C4—C5—C6	120.51 (12)	C17—C20—H20B	109.5
C4—C5—H5	119.7	H20A—C20—H20B	109.5
C6—C5—H5	119.7	C17—C20—H20C	109.5
C5—C4—C3	120.69 (11)	H20A—C20—H20C	109.5
C5—C4—H4	119.7	H20B—C20—H20C	109.5
C3—C4—H4	119.7	C8—C9—C10	118.76 (11)
C19—C17—C18	110.87 (10)	C8—C9—H9	120.6
C19—C17—C20	109.39 (10)	C10—C9—H9	120.6
C18—C17—C20	109.59 (10)	C9—C8—C7	121.31 (10)
C19—C17—P1	110.43 (8)	C9—C8—P1	129.83 (9)
C18—C17—P1	110.18 (8)	C7—C8—P1	108.86 (8)
C20—C17—P1	106.27 (8)	C11—C10—C9	120.21 (11)
C16—C13—C15	109.56 (9)	C11—C10—H10	119.9
C16—C13—C14	109.59 (9)	C9—C10—H10	119.9
C15—C13—C14	109.98 (9)	C10—C11—C12	121.24 (11)
C16—C13—P1	104.76 (7)	C10—C11—H11	119.4
C15—C13—P1	111.52 (8)	C12—C11—H11	119.4
C14—C13—P1	111.29 (8)	Pd1^i^—Cl3—Pd1	94.884 (10)
C12—C7—C8	119.19 (11)	C8—P1—C1	93.92 (5)
C12—C7—C2	126.66 (10)	C8—P1—C17	108.77 (5)
C8—C7—C2	114.12 (10)	C1—P1—C17	109.07 (5)
C17—C19—H19A	109.5	C8—P1—C13	110.90 (5)
C17—C19—H19B	109.5	C1—P1—C13	111.69 (5)
H19A—C19—H19B	109.5	C17—P1—C13	119.48 (5)
C17—C19—H19C	109.5	Cl1—Pd1—Cl2	91.333 (10)
H19A—C19—H19C	109.5	Cl1—Pd1—Cl3^i^	90.924 (10)
H19B—C19—H19C	109.5	Cl2—Pd1—Cl3^i^	177.377 (10)
C17—C18—H18A	109.5	Cl1—Pd1—Cl3	175.919 (10)
C17—C18—H18B	109.5	Cl2—Pd1—Cl3	92.603 (10)
H18A—C18—H18B	109.5	Cl3^i^—Pd1—Cl3	85.117 (10)
C17—C18—H18C	109.5		
			
C5—C6—C1—C2	1.22 (17)	C7—C8—P1—C17	107.86 (8)
C5—C6—C1—P1	−177.59 (9)	C9—C8—P1—C13	60.63 (12)
C3—C2—C1—C6	−1.94 (17)	C7—C8—P1—C13	−118.81 (8)
C7—C2—C1—C6	176.93 (10)	C6—C1—P1—C8	−176.59 (11)
C3—C2—C1—P1	177.11 (9)	C2—C1—P1—C8	4.49 (9)
C7—C2—C1—P1	−4.03 (12)	C6—C1—P1—C17	72.01 (12)
C1—C2—C3—C4	0.77 (17)	C2—C1—P1—C17	−106.92 (8)
C7—C2—C3—C4	−177.94 (11)	C6—C1—P1—C13	−62.25 (12)
C1—C6—C5—C4	0.65 (18)	C2—C1—P1—C13	118.82 (8)
C6—C5—C4—C3	−1.81 (19)	C19—C17—P1—C8	−170.94 (8)
C2—C3—C4—C5	1.08 (18)	C18—C17—P1—C8	66.24 (9)
C3—C2—C7—C12	1.82 (19)	C20—C17—P1—C8	−52.40 (9)
C1—C2—C7—C12	−176.95 (11)	C19—C17—P1—C1	−69.77 (9)
C3—C2—C7—C8	−179.97 (11)	C18—C17—P1—C1	167.42 (8)
C1—C2—C7—C8	1.26 (14)	C20—C17—P1—C1	48.77 (9)
C8—C7—C12—C11	−1.44 (17)	C19—C17—P1—C13	60.37 (10)
C2—C7—C12—C11	176.69 (11)	C18—C17—P1—C13	−62.44 (10)
C10—C9—C8—C7	0.39 (16)	C20—C17—P1—C13	178.91 (8)
C10—C9—C8—P1	−178.98 (9)	C16—C13—P1—C8	52.91 (9)
C12—C7—C8—C9	1.04 (16)	C15—C13—P1—C8	171.33 (8)
C2—C7—C8—C9	−177.32 (10)	C14—C13—P1—C8	−65.44 (9)
C12—C7—C8—P1	−179.47 (9)	C16—C13—P1—C1	−50.42 (9)
C2—C7—C8—P1	2.18 (12)	C15—C13—P1—C1	68.00 (9)
C8—C9—C10—C11	−1.41 (17)	C14—C13—P1—C1	−168.77 (8)
C9—C10—C11—C12	1.01 (18)	C16—C13—P1—C17	−179.38 (7)
C7—C12—C11—C10	0.44 (18)	C15—C13—P1—C17	−60.96 (10)
C9—C8—P1—C1	175.63 (11)	C14—C13—P1—C17	62.27 (9)
C7—C8—P1—C1	−3.81 (8)	Pd1^i^—Cl3—Pd1—Cl2	−178.700 (11)
C9—C8—P1—C17	−72.71 (12)	Pd1^i^—Cl3—Pd1—Cl3^i^	0.0

Symmetry code: (i) −*x*, −*y*, −*z*+2.

### Hydrogen-bond geometry (Å, º)

Cg7 is the centroid of the Pd1,Cl3,Pd1',Cl3' ring.

**Table:**

*D*—H···*A*	*D*—H	H···*A*	*D*···*A*	*D*—H···*A*
C11—H11···*Cg*7^ii^	0.95	2.68	3.5952 (13)	138

Symmetry code: (ii) *x*+1, *y*, *z*−1.

## References

[bb1] Allen, F. H. (2002). *Acta Cryst.* B**58**, 380–388. 10.1107/s0108768102003890 12037359

[bb2] Beletskaya, I. P. & Cheprakov, A. V. (2004). *J. Organomet. Chem.* **689**, 4055–4082.

[bb3] Bruker (2010). *APEX2*, *AXScale* and *SAINT* Bruker AXS Inc., Madison, Wisconsin, USA.

[bb4] Dolomanov, O. V., Bourhis, L. J., Gildea, R. J., Howard, J. A. K. & Puschmann, H. (2009). *J. Appl. Cryst.* **42**, 339–341.

[bb5] Migowski, P. & DuPont, J. (2007). *Chem. Eur. J.* **13**, 32–39. 10.1002/chem.200601438 17115465

[bb6] OrLyé, F. d’ & Jutland, A. (2005). *Tetrahedron*, **61**, 9670–9678.

[bb7] Ormondi, B., Shaw, M. L. & Holzapfel, C. W. (2011). *J. Organomet. Chem.* **696**, 3091–3096.

[bb8] Sheldrick, G. M. (2008). *Acta Cryst.* A**64**, 112–122. 10.1107/S0108767307043930 18156677

[bb9] Spek, A. L. (2009). *Acta Cryst.* D**65**, 148–155. 10.1107/S090744490804362X PMC2631630 19171970

[bb10] Westrip, S. P. (2010). *J. Appl. Cryst.* **43**, 920–925.

[bb11] Williams, D. B. G., Shaw, M. L., Green, M. J. & Holzapfel, C. W. (2008). *Angew. Chem. Int. Ed.* **47**, 560–563. 10.1002/anie.200702889 18041799

